# Association of objectively measured physical fitness during pregnancy with maternal and neonatal outcomes. The GESTAFIT Project

**DOI:** 10.1371/journal.pone.0229079

**Published:** 2020-02-18

**Authors:** Laura Baena-García, Irene Coll-Risco, Olga Ocón-Hernández, Lidia Romero-Gallardo, Pedro Acosta-Manzano, Linda May, Virginia A. Aparicio

**Affiliations:** 1 Department of Nursing, Faculty of Health Sciences, University of Granada, Granada, Spain; 2 Department of Physiology, "José Mataix Verdú" Institute of Nutrition and Food Technology (INYTA) and Biomedical Research Centre (CIBM), University of Granada, Granada, Spain; 3 Sport and Health University Research Institute (iMUDS), University of Granada, Granada, Spain; 4 UGC of Gynaecology and Obstetrics, “San Cecilio” University Hospital, Granada, Spain; 5 Department of Physical Education and Sports, Faculty of Sport Sciences, University of Granada, Granada, Spain; 6 Department of Foundational Science and Research, School of Dental Medicine, East Carolina University, Greenville, NC, United States of America; Mount Sinai Health System, University of Toronto, CANADA

## Abstract

**Aim:**

To analyse i) the association of physical fitness during early second trimester and late pregnancy with maternal and neonatal outcomes; and ii) to investigate whether physical fitness is associated with the type of birth (vaginal or caesarean section).

**Methods:**

Pregnant women from the GESTAFIT Project (n = 159) participated in this longitudinal study. Maternal physical fitness including upper- and lower-body strength, cardiorespiratory fitness (CRF) and flexibility were measured through objective physical fitness tests at the 16^th^ and 34^th^ gestational weeks. Maternal and neonatal outcomes were collected from obstetric medical records. Umbilical arterial and venous blood gas pH and partial pressure of carbon dioxide (PCO_2_) and oxygen (PO_2_), were assessed.

**Results:**

At the 16^th^ week, greater upper-body muscle strength was associated with greater neonatal birth weight (r = 0.191, *p*<0.05). Maternal flexibility was associated with a more alkaline arterial pH (r = 0.220, *p*<0.05), higher arterial PO_2_ (r = 0.237, *p*<0.05) and lower arterial PCO_2_ (r = -0.331, *p*<0.01) in umbilical cord blood. Maternal CRF at the 16^th^ gestational week was related to higher arterial umbilical cord PO_2_ (r = 0.267, *p*<0.05). The women who had caesarean sections had lower CRF (*p<*0.001) at the 16^th^ gestational week and worse clustered overall physical fitness, both at the 16^th^ (-0.227, *p =* 0.003, confidence interval (CI): -0.376, -0.078) and 34^th^ gestational week (-0.223; *p =* 0.018; CI: -0.432, -0.015) compared with the women who had vaginal births.

**Conclusion:**

Increasing physical fitness during pregnancy may promote better neonatal outcomes and is associated with a decrease in the risk of caesarean section.

This trial was registered at ClinicalTrials.gov (NCT02582567) on October 20, 2015.

## Introduction

Research continues to confirm that exercise during pregnancy contributes to healthier outcomes for both the mother and the fetus [1,2]. Consequently, exercise is currently recommended in all low risk pregnancies [1]. Exercise during pregnancy has been related to an enhanced efficiency of the placenta [3] and the placental uterine perfusion [4], a lower rate of caesarean sections [1], and optimal birth weight [5]. Improved physical fitness is usually observed in women who exercise during pregnancy [6] and is associated with better perinatal health outcomes [7,8]. However, while it is well-established that physical activity improves birth outcomes [9–11], studies looking at the relationships between objective assessments of fitness and delivery type, gestational age, duration of labour stages, birth weight and values of umbilical cord blood gas are limited.

The causes of caesarean sections and how to reduce caesarean section rates are now a spotlight of research as this type of delivery has been associated with an increased risk of infection compared with vaginal deliveries, higher wound dehiscence, haemorrhage and reduced fertility [12–14], as well as an increased risk of allergies, type 1 diabetes or respiratory problems [15] in infants, among other complications. Furthermore, it is noteworthy that in 2016 in Spain there were 22% caesarean sections in public hospitals [16], higher than the 15% rate recommended by the World Health Organization [17]. Therefore, it is of great importance for clinical practice to analyse the associations between maternal variables and improvements in the rates of caesarean sections.

During pregnancy, uterine blood flow is crucial for appropriate nutrient and gas exchange with the fetus [18]. It is widely known that the umbilical cord blood pH analysis provides vital information about neonatal health status and reflects physiological response to complications that may occur during labour [19]. We previously found that higher levels of light or moderate physical activity during pregnancy were associated with greater umbilical arterial oxygen saturation and higher levels of venous cord blood pH; conversely, we showed that higher levels of sedentary time during pregnancy were related to worse pregnancy outcomes [10]. However, it is unknown whether maternal physical fitness is also a key factor to improve birth outcomes such as gestational age, duration of labour, birth weight and values of umbilical cord blood gas. This study highlights the importance of appropiate levels and quality of fitness that should be emphazised in exercise programs in order to improve birth outcomes. Furthermore, the association of maternal physical fitness with fetal acid-base balance has not been studied. The measurement of gases in the umbilical cord blood is a gold standard in the determination of the foetal acid-base balance [20]. In addition, the determination of PCO_2_ is important, since higher levels of PCO_2_ and a low pH level indicate a state of fetal acidosis [19,21]. On the other hand, CO_2_ production is proportional to oxygen consumption [22], so the interpretation of PO_2_ levels and oxygen saturation also provides fundamental information to determine the effectiveness of the fetal compensatory mechanisms and the state of the placenta. Therefore, the aims of this study were: i) to analyse the association of maternal physical-fitness measures during the early second trimester and late pregnancy with maternal (gestational age at birth and length of the first and second stages of labour) and neonatal (birth weight and umbilical cord blood gas values) outcomes; ii) to investigate whether maternal physical-fitness during the early second trimester and late pregnancy may influence the type of birth (i.e. vaginal or caesarean section). Accordingly, we hypothesized that maternal physical-fitness may be positively associated with improved maternal and neonatal outcomes. The aim of the study was to better understand the influence of maternal fitness during pregnancy on maternal and neonatal outcomes.

## Materials and methods

### Study population

The detailed procedures, inclusion and exclusion criteria (**S1 Table**) of the GESTAtion and FITness (GESTAFIT) Project were published elsewhere [23]. Initially, this study was based on a randomized control trial design, that was modified in order to ensure retention of women in the control group. Thus, women were allocated either to an exercise or a control group depending on their personal preference and convenience to attend the intervention sessions and the wave they had been recruited for. In this way, high dropout rates were avoided, which is one of the most frequent methodological barriers in antenatal exercise research, as previously argued [24]. Briefly, the intervention group underwent a supervised aerobic and strength training intervention from the 17^th^ gestational week until delivery. A total of 384 pregnant women were informed about the Project during their 12^th^ gestational week visit to the obstetrician at the “San Cecilio” University Hospital and “Virgen de las Nieves” University Hospital (Granada, southern Spain). A final number of 159 women were interested in participating and signed an informed consent. The GESTAFIT study was approved by the Ethics Committee on Clinical Research of Granada, Government of Andalusia, Spain (code: GESTAFIT-0448-N-15, approved on 19/05/2015).

### Procedures

The first evaluation of the study was completed at the 16^th^ week of gestation (±2 weeks). Women completed a self-reported questionnaire, an anthropometric assessment, and physical fitness tests. On the 34^th^ week of gestation (±2 weeks), the second assessment of physical fitness tests and height and weight was conducted. Birth and obstetric outcomes of the current pregnancy were collected from the digital medical records (the pregnancy health document and the partogram). Sampling of the umbilical cord occurred 3 minutes after birth.

### Measurements

#### Maternal sociodemographic data

Sociodemographic data (age, number of children; marital, educational and working status) were assessed with a self-reported questionnaire. A research staff was present at all times for any needed explanation or instruction.

#### Maternal anthropometric assessment

Height and weight were measured using a stadiometer (Seca 22, Hamburg) and a scale (InBody R20; Biospace, Seoul, Korea), respectively. Body mass index was calculated as: weight (Kg)/height (m^2^).

#### Pregnancy health

The “Pregnancy Health Document” (*Cartilla del embarazo* in Spanish) is given to all pregnant women by the Andalusian regional government, and it contains obstetric and medical data recorded during all the length of the pregnancy and were collected for the present study. In this way, information about previous pregnancies, births, and gynaecological antecedents were obtained. The gestational age was calculated by the date of the last menstruation corrected for cycles of 28 days and subsequently corrected by ultrasound, if needed [25], and was used for an accurate timing of the evaluations at the 16^th^ and the 34^th^ weeks of pregnancy.

#### Birth outcomes

All data related to the type of birth (spontaneous vaginal, instrumental or caesarean), gestational week at birth, use of epidural analgesia, length of each stage of labour, offspring sex, neonatal weight and Apgar test were obtained from perinatal obstetric records (*partogram*) from the Hospital after birth. The cause (unplanned or planned, and whether it was elected for physiological reasons) of caesarean sections was annotated. We only had access to the partograms and samples of women who gave birth in the Public Hospitals approved by the Ethics Committee. However, the type of delivery was recorded for all women.

The placenta was collected immediately after the delivery and the placental surface clots were removed. The amnion was cut from the basal area and the umbilical cord was completely cut. Subsequently, placenta was weighted with a 3.200 kg Precision Gram Scale (Ohaus Compass^TM^, USA).

#### Umbilical cord blood gas

For the umbilical cord blood sampling, a trained midwife performed a double clamping of the umbilical cord three minutes after birth, with a minimum distance between both clamps of 10 centimetres. A pre-heparinized 1mL syringe was used for blood extraction. Blood samples were taken from both, umbilical artery and vein. Partial pressure of carbon dioxide (PCO2), partial pressure of oxygen (PO2), oxygen saturation and pH were analysed using a blood analyser (GEM Premier 4000; Instrumentation Laboratory, Bedford, MA, USA).

#### Physical fitness

Physical fitness was assessed in the following order to avoid potentially induced fatigue from other tests:

*Upper-body muscular strength*. Hand grip strength was measured with digital dynamometry (TKK 5101 Grip-D; Takey, Tokyo, Japan) after adjusting for the hand size for an optimal grip [26]. The test was performed twice with both hands, with a rest of 30 seconds and alternating hands. The final value was calculated as a mean of the two values from each hand.

*Lower-body muscular strength*. Muscular strength of the lower-body was measured through the 30-second Chair Stand test. This test counts the maximum number of repetitions completed in 30 seconds, with a full stand to a sitting position and back straight up as one repitition. The participants performed one trial after a familiarization trial [27].

*Flexibility*. The Back Scratch test was used to assess upper-body flexibility. The test consists in measuring the overall shoulder range of motion by measuring the distance between the middle fingers coming together behind the back. The Back Scratch test outcome is positive for higher flexibility (i.e. hands overlapping behind the back) and negative for lower flexibility (i.e. greater distance between middle fingers behind the back). This test was done twice with both hands and the final score was calculated as the mean between the two attempts of each arm [27].

*Cardiorespiratory fitness*. Maximal oxygen intake (VO_2max_) was estimated (ml/Kg/min) through the Modified Bruce treadmill protocol [28], a submaximal, incremental, multistage, continuous treadmill test. The test consists of progressive increments in the workload and velocity every 3 minutes to determine limits of maximal exertion. Women were asked to walk on the treadmill during the test until the maternal heart rate reached 75% of the age-predicted maximal heart rate; if the participant requested to end the treadmill test, then the test was also stopped before reaching the heart rate value. Although submaximal treadmill testing is common and safe during pregnancy, women were secured with a harness during the test to prevent any consequences from a fall. This test has been previously used and shown to be safe in pregnant women [28,29].

#### Statistical analyses

We employed descriptive statistics [mean (standard deviation, SD)] for quantitative variables and number of cases and percentage (%) for categorical variables. All the variables were checked for normality of distribution through kurtosis and skewness analyses before the analyses. All the outcome variables reasonably met this assumption. The associations of physical fitness levels with maternal (gestational age at birth and length of the first and second stages of labour) and neonatal (birth weight and umbilical cord blood gas values) outcomes were assessed with partial correlations after adjusting for maternal age, parity and BMI (Table 2). The associations of physical fitness levels with duration of the first and second labour stages, as well as neonatal outcomes were also adjusted for epidural analgesia, except for the variable “birth weight” which was only further adjusted for gestational age. Since some variables (gestational age at birth, birth weight, duration of the first and second stages of labor and instrumental delivery) previously showed weak associations with outcome variables (*p*>0.02), we performed secondary analyses to assess their role as potential confounders.

Changes between pre (16^th^ week) and post (34^th^ week) evaluations results of fitness tests were analysed via repeated measures T-Tests (**S2 Table**). Subsequently, the changes (post–pre) were included in the linear regression analyses (**Table 3**) as predictor variables, whereas the birth outcomes were included as outcome variables in separate models. Relevant confounders suggested by previous literature with significant relationship with the outcomes, that influenced the relationship between the independent and dependent variable (i.e. B of the independent had a meaningful change (*p*>0.05) when adding the variable), were included in the models. Depending on the aim of the analysis, the models were adjusted for none confounder, for baseline values of cardiorespiratory fitness (CRF), or for baseline values, age, parity, weight change (post–pre), and exercise intervention (only at the 34^th^-week analysis). The variables showing the duration of the first and second labour stages, as well as neonatal birth outcomes were additionally adjusted for epidural analgesia (as independent variable), except for the variable “birth weight” which was only further adjusted for gestational age.

Logistic regression analysis was also employed to explore the associations between physical fitness levels (predictor variable) and the chances of having a vaginal delivery (spontaneous and instrumental) or caesarean section (outcome variable) (**Table 4**). We adjusted the analysis for the above-mentioned covariates. We also considered “place of birth” as a potential confounder due to the differences of caesarean section ratios between private and public hospitals in Spain [30]. Hence, we decided to check with the Chi-squared test if the type of hospital might have any influence on outcome variables. Given that we found statistically significant differences in birth outcomes between private and public hospitals within our data, we decided to include this variable as a potential confounder.

Finally, in the GESTAFIT project [23], a concurrent physical exercise program was performed. We have also adjusted all the models for the exercise intervention (control or intervention).

We created a clustered overall physical fitness (Z-score) as the mean of the standardized scores [(value–mean)/ standard deviation] of body strength (mean of upper and lower- body strength standardized values), flexibility and CRF at the 16^th^ week of pregnancy (**Fig 2A**) and the 34^th^ week of pregnancy (**Fig 2B**). Both clustered groups were compared by caesarean or vaginal delivery with an ANCOVA analysis. We adjusted the analysis for maternal age, parity, maternal BMI at the 16^th^ gestational week, exercise intervention and birth place (public or private hospital).

The statistical analyses were conducted with the Statistical Package for Social Sciences (IBM SPSS Statistics for Windows, Version 20.0. Armonk, NY: IBM Corp). The statistical significance was set at *p*<0.05.

## Results

**Fig 1:**
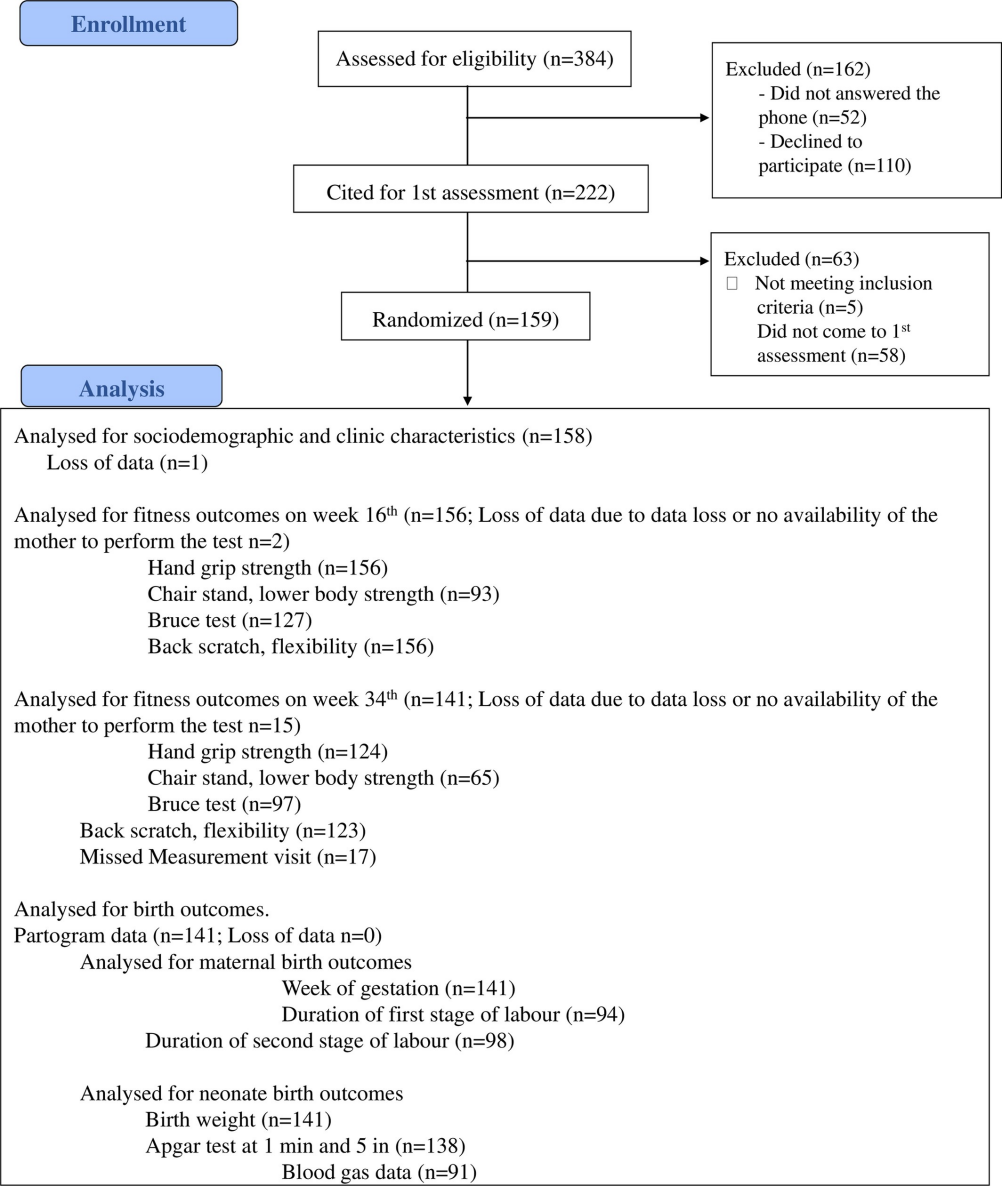
Flowchart in the selection of study participants for specific study aims.

Of all the 159 participants, 158 had complete sociodemographic data (**Fig 1**). The final sample size was composed of 158 Caucasian pregnant women (age 32.9±4.6 years, BMI 24.9±4.1 kg/m^2^) with valid data for the present analyses. At the 16^th^ and 34^th^ weeks of pregnancy, 156 and 124 women participated in the physical fitness tests (**Fig 1**). A total of 18 partograms were not found in the informatic system (n = 9) or could not be collected as some participants delivered in a private hospital not associated with the study (n = 9) and partograms could not be accessed, neither could the samples of placenta be collected. However, the type of delivery was recorded for the deliveries in private hospitals. Hence, a total of 141 women had complete valid data for birth outcomes.

The sociodemographic and clinical characteristics of the study participants are shown in **Table 1**. Most of the participants lived with their partner and more than half had University education and worked full time. Approximately 61% of the sample were nulliparous and 75.6% had vaginal births. Births took place around 39.5±1.3 weeks of gestation, with a mean neonate body weight of 3305±480.6 grams. At the 16^th^ and 34^th^ gestational weeks, mean values were similar in upper-body strength (Hand grip); lower-body strength (Chair stand); and 4.1±6.2 cm and 3.9±6 cm in flexibility test (Back Scratch) (all, *p*>0.05), but decreased in CRF 22.1±5.5 ml/Kg/min and 17.9±4.9 ml/Kg/min (*p*<0.001) (Modified Bruce treadmill test). All umbilical cord blood gas mean values were within normal ranges.

**Table 1:** Sociodemographic and clinical characteristics of the study sample (n = 158).

Maternal characteristics	n	Mean (SD)
Age, years	158	32.9 (4.6)
Body mass index at the 16^th^ gestational week, Kg/m^2^	158	24.9 (4.1)
Body mass index at the 34^th^ gestational week, Kg/m^2^	158	27.8 (4.1)
Weight at the 16^th^ gestational week, Kg	157	67.04 (11.8)
Weight at the 34^th^ gestational week, Kg	123	74.57 (10.8)
Weight change (from the 16^th^ to the 34^th^ gestational weeks)	121	8.7 (3.4)
Ethnicity (Caucasian)	158	158 (100)
		**n (%)**
Living with a partner,	154	(97.5)
Educational status	158	
*Primary or high-school*		37 (23.4)
*Specialized training*		27 (17.1)
*University degree*		94 (59.5)
Working status	158	
*Homework/unemployed*		48 (30.4)
*Partial-time employed/student*		41 (25.9)
*Full-time employed*		69 (43.7)
Type of birth	143	
*Spontaneous*		83 (58)
*Instrumental vacuum/forceps*		24 (16.8)
*Caesarean section*		36 (25.5)
*Planned (feet or buttocks coming first)*		7 (27.5)
Birth place	147	
*Public Hospital*		138 (93.9)
*Private Hospital*		8 (5.4)
*Home*		1 (0.7)
Parity	158	
*Nulliparous*		96 (60.8)
*Multiparous*		62 (39.2)
Physical fitness at the 16^th^ gestational week		**Mean (SD)**
*Upper-body strength (Hand grip)*, *mean (Kg)*	156	27.2 (4.2)
*Lower-body strength (Chair stand)*, *number of repetitions*	93	15.7 (2.4)
*Flexibility (Back Scratch)*, *mean (cm)*	156	4.1 (6.2)
*CRF (Modified Bruce test)*, *VO*_*2max*_	127	22.2 (5.5)
Physical fitness at the 34^th^ gestational week		
*Upper-body strength (Hand grip)*, *mean (Kg)*	124	27.2 (4.4)
*Lower-body strength (Chair stand)*, *number of repetitions*	65	15.9 (2.5)
*Flexibility (Back Scratch)*, *mean (cm)*	123	3.9 (6)
*CRF (Modified Bruce test)*, *VO*_*2max*_	97	17.9 (4.9)
**Neonatal outcomes**		
Sex (female, n (%))	141	72 (51.4)
Gestational age at birth, wk	141	39.5 (1.3)
Birth weight, grams	141	3305 (480.6)
Placental weight, grams	106	573.7 (102.6)
Apgar score 1 minute	138	8.6 (1)
Apgar score 5 minutes	138	9.6 (0.7)
Umbilical Cord blood Gas		
*Arterial pH*	103	7.2 (0.07)
*Arterial Partial Pressure CO*_*2*_, *mmHg*	97	51.1 (10.3)
*Arterial Partial Pressure O*_*2*_, *mmHg*	92	19.8 (8.9)
*Arterial O*_*2*_ *saturation*, *%*	90	35.6 (21.8)
*Venous pH*	117	7.3 (0.1)
*Venous Partial Pressure CO*_*2*_, *mmHg*	112	39.7 (7.7)
*Venous Partial Pressure O*_*2*_, *mmHg*	102	26.3 (9.2)
*Venous O*_*2*_ *saturation*, *%*	97	54.9 (18.4)

Values shown as mean (SD, standard deviation) unless otherwise indicated; BMI, body mass index; CRF, cardiorespiratory fitness; CO_2,_ carbon dioxide; O_2_, oxygen.

Pearson’s partial correlations of physical fitness levels at the 16^th^ and the 34^th^ gestational weeks with maternal and neonatal outcomes are shown in **Table 2.** Regarding the maternal outcomes, physical fitness levels were not associated with these measures. In relation to neonatal outcomes, upper-body muscle strength at the 16^th^ week of gestation was associated with higher birth weight (r = 0.191, *p*<0.05). Lower-body muscle strength was not associated with any maternal and neonatal outcome (*p*>0.05). Greater maternal flexibility at the 16^th^ week was associated with a more alkaline pH (r = 0.220, *p*<0.05), higher PO_2_ (r = 0.237, *p*<0.05), higher arterial oxygen saturation (r = 0.242, *p*<0.05), and lower PCO_2_ (r = -0.331, *p*<0.01) in arterial umbilical cord blood. Maternal CRF at the 16^th^ week was related to higher PO_2_ (r = 0.267, *p*<0.05) and higher oxygen saturation (r = 0.375, *p*<0.01) in arterial umbilical cord blood. Physical fitness levels at the 34^th^ week of pregnancy were not associated with any maternal and neonatal birth outcomes (all, *p* >0.05).

**Table 2:** Partial correlations of physical fitness levels at the 16^th^ and the 34^th^ weeks gestation with maternal and neonatal outcomes.

Maternal outcomes		Upper-body muscle strength	Lower-body muscle strength	Flexibility	Cardiorespiratory fitness
Week of gestation (at birth)	16 g.w.	.008 (n = 122)	.163 (n = 66)	-.035 (n = 121)	.072 (n = 98)
	34 g.w.	.052 (n = 103)	.040 (n = 49)	-.034 (n = 102)	-.086 (n = 76)
Duration of the first stage of labour ^a^	16 g.w.	.200 (n = 81)	.136 (n = 42)	-.037 (n = 81)	-.120 (n = 66)
	34 g.w.	.132 (n = 65)	.085 (n = 28)	.023 (n = 64)	-.046 (n = 46)
Duration of the second stage of labour ^a^	16 g.w.	-.149 (n = 84)	-.011 (n = 45)	-.015 (n = 84)	-.095 (n = 69)
	34 g.w.	-.097 (n = 70)	-.166 (n = 32)	-.008 (n = 69)	.016 (n = 50)
**Neonatal outcomes** ^a^					
Birth weight	16 g.w.	.191* (n = 122)	.090 (n = 66)	.050 (n = 121)	-.077 (n = 98)
	34 g.w.	.139 (n = 103)	-.059 (n = 49)	.103 (n = 102)	-.076 (n = 76)
Cord blood arterial pH	16 g.w.	-.048 (n = 83)	.175 (n = 42)	.220* (n = 82)	.084 (n = 65)
	34 g.w.	.095 (n = 70)	-.115 (n = 31)	.113 (n = 69)	.079 (n = 54)
Cord blood arterial PCO_2_	16 g.w.	-.056 (n = 78)	-.103 (n = 37)	-301**(n = 77)	-.140 (n = 60)
	34 g.w.	-.096 (n = 66)	.260 (n = 28)	-.220 (n = 65)	-.070 (n = 52)
Cord blood arterial PO_2_	16 g.w.	.104 (n = 74)	-.091 (n = 38)	.237* (n = 73)	.267* (n = 60)
	34 g.w.	.016 (n = 62)	-.188 (n = 29)	.162 (n = 62)	-.182 (n = 48)
Cord blood arterial O_2_ saturation	16 g.w.	.015 (n = 72)	-.066 (n = 36)	.242* (n = 71)	.372**(n = 57)
	34 g.w.	.016 (n = 61)	-.265 (n = 27)	.162 (n = 60)	-.014 (n = 47)
Cord blood venous pH	16 g.w.	.089 (n = 95)	.110 (n = 47)	.144 (n = 94)	.062 (n = 76)
	34 g.w.	.204 (n = 81)	.099 (n = 36)	.102 (n = 80)	.041 (n = 63)
Cord blood venous PCO_2_	16 g.w.	-.113 (n = 91)	-.058 (n = 43)	-.0158 (n = 90)	-.036 (n = 72)
	34 g.w.	-.189 (n = 78)	-.154 (n = 34)	-.030 (n = 77)	.013 (n = 62)
Cord blood venous PO_2_	16 g.w.	-.015 (n = 83)	.056 (n = 41)	.129 (n = 82)	-.018 (n = 65)
	34 g.w.	.032 (n = 69)	.167 (n = 32)	.086 (n = 68)	-.160 (n = 53)
Cord blood venous O_2_ saturation	16 g.w.	.129 (n = 73)	.185 (n = 38)	.123 (n = 77)	0.15 (n = 62)
	34 g.w.	.030 (n = 70)	.221 (n = 29)	.032 (n = 65)	-.116 (n = 52)

O_2_, Oxygen; PO_2,_ partial pressure of oxygen; PCO_2,_ partial pressure of carbon dioxide; g.w, gestational week. Model adjusted for age, parity, maternal body mass index, gestational week, and the exercise intervention. Maternal birth outcomes were additionally adjusted for birth weight.

^a^ Model additionally adjusted for epidural analgesia. Birth weight was further adjusted for gestational age.

*p<0.05

**p<0.01.

**S2 Table**, comparing the fitness levels at the 16^th^ and the 34^th^ weeks of gestation, there were no differences in flexibility, upper- or lower-body strength between the different time points. CRF decreased by 4.14 (5.07) ml/kg/min from the 16^th^ to the 34^th^ weeks of gestation (95% CI: 3.01, 5.27; *p<*0.001).

The associations of the changes in CRF with birth outcomes are shown in **Table 3**. Arterial cord blood PO_2_ and O_2_ saturation were inversely associated with the CRF change (B = -0.603; 95% CI: -1.101, -0.105; p = 0.019, and B = -1.544; 95% CI: -2.720, -0.368; p = 0.011, respectively), with similar results in the model adjusted for baseline CRF, age, parity, weight change and exercise intervention. The model adjusted only for baseline values of CRF showed an inverse association between CRF change and arterial cord blood PO_2_ (B = -0.622; 95% CI: -1.221, -0.023; p = 0.042).

**Table 3:** Associations of changes in cardiorespiratory fitness from the 16^th^ to 34^th^ weeks of gestation with outcomes.

	Unadjusted			Model 1			Model 2		
Maternal outcomes	B	95% CI	p	B	95% CI	p	B	95% CI	p
Week of gestation (at birth)	-0.029	(39.26, 39.90)	0.235	-0.049	(-0.111, 0.012)	0.113	-0.068	(-0.137, 0.001)	0.052
Duration of the first stage of labour ^a^	4.018	(-5.35, 13.38)	0.393	1.545	(-11.35, 14.44)	0.811	3.826	(-5.741, 13.393)	0.423
Duration of the second stage of labour ^a^	2.270	(-1.44, 5.98)	0.226	2.787	(-1.811, 7.385)	0.230	0.894	(-3.116, 4.904)	0.656
**Neonatal outcomes**									
Birth weight	5.607	(-13.35, 24.56)	0.557	0.861	(-22.82, 21.54)	0.942	20.176	(-4.763, 45.115)	0.111
Cord blood arterial pH ^a^	0.001	(-0.004, 0.005)	0.773	0.001	(-0.004, 0.006)	0.789	-0.001	(-0.007, 0.005)	0.673
Cord blood arterial PCO_2_ ^a^	0.130	(-0.449, 0.710)	0.653	0.152	(-0.538, 0.842)	0.661	0.208	(-0.628, 1.044)	0.619
Cord blood arterial PO_2_ ^a^	-0.603	(-1.101, -0.105)	0.019	-0.622	(-1.221, -0.023)	0.042	-0.960	(-1.659, -0.260)	0.009
Cord blood arterial O_2_ saturation ^a^	-1.544	(-2.720, -0.368)	0.011	-1.249	(-2.665, 0.167)	0.082	-2.428	(-3.907, -0.650)	0.007
Cord blood venous pH ^a^	0.001	(0.002, 0.004)	0.536	0.001	(0.003, 0.005)	0.602	0.000	(-0.005, 0.004)	0.905
Cord blood venous PCO_2_ ^a^	-0.680	(-0.417, 0.282)	0.699	-0.024	(-0.481, 0.433)	0.917	0.095	(-0.426, 0.616)	0.717
Cord blood venous PO_2_ ^a^	0.154	(-0.266, 0.575)	0.465	-0.071	(-0.632, 0.491)	0.802	0.202	(-0.837, 0.432)	0.523
Cord blood venous O_2_ saturation ^a^	0.104	(-0.732, 0.940)	0.803	-0.350	(-1.507, 0.806)	0.546	-1.081	(-2.363, 0.200)	0.096

Model 1 was adjusted for the baseline value of cardiorespiratory fitness; Model 2 was adjusted for the baseline value of cardiorespiratory fitness, maternal age, parity, weight change and exercise intervention; maternal birth outcomes were additionally adjusted for birth weight.

^a^ Model additionally adjusted for epidural analgesia. Birth weight was further adjusted for gestational age.

Differences in physical fitness levels at the 16^th^ and 34^th^ gestational weeks by birth type (vaginal or caesarean section) are shown in **Table 4**. At the 16^th^ week of pregnancy, no significant differences were found in upper- and lower-body muscle strength between women who had vaginal births compared with women who had caesarean sections (both, *p*>0.05). The mean of flexibility levels was +2.4 (4.7) cm in the Back Scratch test in women who had caesarean sections compared with +5.0 (6.2) cm in women who had vaginal births (95% CI -0.027, -0.020; *p* = 0.027). Finally, women who had caesarean sections had a mean of CRF of 18.4 (4.2) ml/kg/min in the Modified Bruce test compared with women with a vaginal birth who had 23.3 (5.7) ml/kg/min in CRF (95% CI: -0.035, -0.009; *p =* 0.001). For physical fitness at the 34^th^ week of gestation, flexibility was higher in women who had vaginal births with 4.9 (6.0) cm, than in women with caesarean section, 1.0 (5.8) cm (95% CI: 0.027, 0.001; *p =* 0.033).

**Table 4:** Differences in physical fitness of the pregnant women at the 16^th^ and the 34^th^ weeks gestation by birth type.

		Vaginal	Caesarean	B	95% CI	*p*
Upper-body muscle Strength, kg	16 g.w.	27.6 (4.1) n = 102	26.8 (3.7) n = 30	-0.013	(-0.031, 0.005)	0.160
	34 g.w.	27.6 (3.7) n = 91	26.5 (4.3) n = 26	-0.014	(-0.033, 0.005)	0.119
Lower-body muscle Strength, kg	16 g.w.	16.1 (2.5) n = 59	15.3 (2.1) n = 16	-0.027	(-0.070, 0.015)	0.208
	34 g.w.	16 (2.6) n = 50	15.5 (2.2) n = 9	-0.018	(-0.057, 0.020)	0.426
Flexibility, cm	16 g.w.	5.0 (6.2) n = 102	2.4 (4.7) n = 29	-0.014	(-0.027, -0.002)	**0.027**
	34 g.w.	4.9 (6.0) n = 89	1.0 (5.8) n = 26	-0.013	(-0.027, -0.001)	**0.033**
Cardiorespiratory fitness, VO_2max_	16 g.w.	23.3 (5.7) n = 86	18.4 (4.2) n = 22	-0.022	(-0.035, -0.009)	**0.001**
	34 g.w.	18.4 (4.9) n = 72	15.7 (4.8) n = 20	-0.015	(-0.032, 0.002)	0.081

CI, Confidence interval; g.w., gestational week. Data adjusted for maternal age, parity, maternal body mass index, exercise intervention and birth place. Values shown as mean (standard deviation).

**Fig 2:**
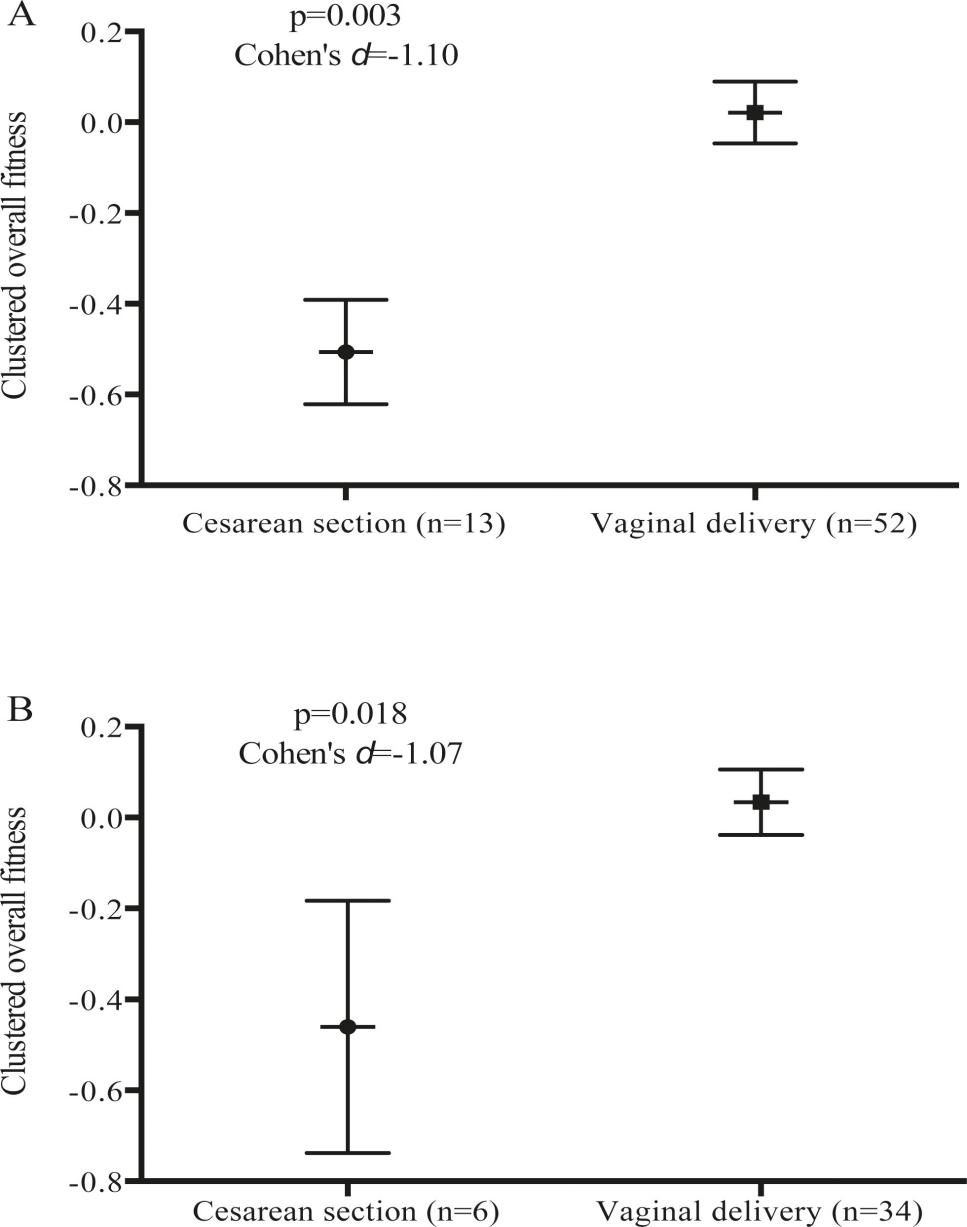


**Fig 2A** and **Fig 2B** show the clustered overall physical fitness at the 16^th^ and 34^th^ weeks of gestation, respectively. Relative to women who had vaginal births, women who had caesarean sections presented worse clustered overall physical fitness at the 16^th^ (mean difference -0.227, *p =* 0.003; 95% CI: -0.376, -0.078) and 34^th^ weeks of gestation (mean difference -0.223, *p =* 0.018; 95% CI: -0.432, -0.015). Results were similar when elective caesarean sections were excluded from the analysis.

## Discussion

To the best of our knowledge, this is the first study assessing the association of objectively measured physical fitness during pregnancy with maternal and neonatal outcomes. One of the major findings of this study is that women who had caesarean deliveries also had decreased CRF, flexibility, and clustered overall physical fitness at 16^th^ and 34^th^ weeks of pregnancy compared with women who had vaginal births. Although no differences were seen in maternal outcomes, the 16^th^ week upper-body strength was related to greater birth weight, and the 16^th^ week maternal CRF and flexibility were positively associated with PO_2_ and oxygen saturation in the arterial umbilical cord.

### Influence of physical fitness on delivery type

In this study, women who had caesarean sections had lower flexibility and CRF and worse clustered overall physical fitness during pregnancy than women who had vaginal births. Regarding the association of flexibility with birth type, it can be explained via the vasodilator effects of relaxin [31], which may promote a better blood flow to the fetus and decrease alterations in fetal well-being. This is a novel finding, since there are no studies that directly relate maternal flexibility to the type of delivery, but exercise during pregnancy such as yoga that includes flexibility training has been associated with less labour pain, shortening of labour and lower caesarean sections [32].

Moreover, regarding CRF, the relation between a higher maternal VO_2max_ and better values of oxygen in the umbilical artery blood as well as a lower rate of caesarean section has never been reported. Labour is a major physical effort that often lasts longer than 7 hours [33]. Therefore women with lower CRF and lower respiratory and circulatory capacity to supply oxygen to the body during a strenuous work probably have a higher prevalence of caesarean delivery.

Finally, the findings about the lower caesarean rate in women with better clustered overall physical fitness during pregnancy are extremely important. Caesarean deliveries are associated with a greater number of postpartum complications for the mother and the neonate [15,34]. More than one in four of the births in our study sample were by caesarean delivery, a much higher rate than the one proposed by the World Health Organization, which establishes that rates above 15% do not reduce maternal and neonatal morbidity and mortality [17]. It must be taken into consideration that in Spain the caesarean rate in private hospitals is 15% higher than in public hospitals [30]. However, the results of the present study are encouraging. Lower rates of caesarean sections and better neonatal outcomes could be promoted by increasing maternal physical fitness, which is also associated with better health of women and offspring in the future.

### Association of maternal physical fitness with birth weight

Our findings of increased maternal upper-body muscle strength at the 16^th^ week of gestation associated with greater neonate birth weight is in agreement with Bisson et al. [35]. It must be highlighted that handgrip strength provides useful information about overall muscular strength [36,37]. On the one hand, increased upper-body muscle strength could be related to an increased risk of fetal macrosomia (birth weight greater than four kilograms) [38]. In this study, only five neonates (3.5%) were macrosomic and among them, only two of the mothers showed higher handgrip strength than the mean of the sample (27.3±4.2 Kg). Therefore, maternal upper-body muscle strength does not seem to be related to a pathological increase in birth. Importantly, Dodds et al. [39] found that neonates with higher birth weight had more muscle strength later in childhood. Thus, the increases in birth weight may be due to increases in neonatal muscle mass. The mechanisms that may explain the relationship between maternal muscle strength and the newborn body weight are still unclear but could be related to changes in placental function. Potentially, maternal exercise may stimulate the placenta to increase IGF production, which then stimulates fetal increases in skeletal muscle mass [40]. Moreover, Lewis et al. [41] reported that women with more arm muscle area also had increased placental amino acid transport, which is essential for appropriate fetal development [41]. This finding could be explained by a placental metabolic change, perhaps due to the need to preserve maternal nutrients in mothers with lower muscle mass and, therefore, lower protein deposits [41]. Another possible explanation for the change in birth weight is increased placental angiogenesis, as it occurs in pregnant women who perform exercise [4]. Further research will need to be done with more specific and longitudinal body composition analyses of offspring in order to determine if maternal physical fitness leads to healthier weights in offsprings at later stages of life [36].

### Association of maternal physical-fitness measures with umbilical cord gases

Since the 16^th^ week measures of CRF and flexibility were positively associated with arterial umbilical cord blood gas, these may relate to the status of connective tissue, i.e. ligaments, during pregnancy. A possible explanation for this association would be the elevated serum relaxin levels during pregnancy [42], which increases ligament laxity [43], necessary for the correct maintenance of pregnancy and the labour progression [44]. In addition, this hormone has a positive effect on uterine and placental growth [45]. For this reason, we hypothesize that the association between flexibility at the 16^th^ gestational week and better umbilical cord blood gas could have a relation with increased levels of relaxin in pregnant women with greater flexibility [46]. Since relaxin has endothelium-dependent vasodilation effects on the uterine artery [31], it seems feasible that the uteroplacental flow was more efficient during the labour in women with higher relaxin. Another plausible reason is that women with greater flexibility have greater laxity of the pelvic ligaments, which increases the degree of bone movement and pelvic diameters [47] and this could favor better birth outcomes. This result is clinically important since lower arterial pH levels (pH<7.20) in the umbilical cord blood are strongly associated with increased neonatal morbidity and mortality [48]. Mukai et al. [49] measured the oxygen saturation in the cranial tissue during the birth crowning and five minutes after birth. The authors described that fetuses with better oxygen saturation during labour also had better oxygen saturation at five minutes after birth [49]. This result, as well as the higher levels of arterial PO_2_ in neonates of mothers with greater flexibility, may be related to a better blood perfusion from the mother to the fetus. This possibility is in agreement with Ramírez et al. [3] who found that exercise, which is closely related to physical fitness, during pregnancy decreased placenta weight, but increased its efficiency. This fact could also explain the positive association observed between maternal VO_2max_ and PO_2_ and oxygen saturation in the arterial umbilical cord. A higher VO_2max_ means a greater capacity of the cardiorespiratory system to supply, transport and optimize the blood flow of oxygen and is usually observed in more physically active people [50]. Thus, it is plausible that this would provide increased oxygen to the fetus. Indeed, placental changes expressed in active pregnant women are associated with improvements in placental perfusion [4]. However, more studies are needed to verify this association.

Despite all these positive findings, the fact that physical fitness at the 34^th^ gestational week was not associated with any birth outcome must be highlighted. It must be considered that participants showed worse physical fitness at the 34^th^ gestational week than at the 16^th^ gestational week, especially regarding CRF, which was much lower. These results are in agreement with previous literature [51,52]. We hypothesize that the increase of uterine size could influence the performance of the tests being the women heavier at the 34^th^ week of pregnancy, and therefore more uncomfortable during the tests [53]. In this regard, other studies using the squat test (similar to the chair stand test) already found a reduction in the ability to perform the test in later stages of pregnancy [54,55]. Regarding the values for CRF, some hypotheses that could explain the low values obtained in the present study sample are: i) the use of submaximal tests, that estimate the effort based on the heart rate; and ii) the usual false belief that women should not exercise when they are pregnant, that seems to be more evident at late gestation, this may limit women to try harder in the tests [56] iii) later in pregnancy women already have an increased resting heart rate, which can influence results [2,57], iv) due to the increased weight, women are also moving at a higher energy cost later in pregnancy relative to a similar treadmill earlier in pregnancy; they will have a higher rate of perceived exertion later in pregnancy and, thus, want to stop the treadmill test earlier when they are further along in pregnancy [58]. In addition, there are still no normal distribution curves that determine the appropriate parameters of PF over gestational age. Therefore, the validation of physical fitness tests for women in late pregnancy would be useful given the anatomical differences and capacities between early and late pregnancy, and the results associated with those changes. Furthermore, most associations in this study were observed at the first assessment, prior to the exercise intervention. Although current literature highlights the importance of physical exercise during pregnancy, the current findings suggest the importance of physical fitness prior to pregnancy. Moreover, the association between physical fitness levels in the early second trimester and some neonatal outcomes may be due to changes in the placental physiology, which predominantly occurs in the first trimester [59]. Therefore, more studies are needed to contrast if this finding is due to the effect of pre-pregnancy physical fitness or placental changes that occur in the first trimester of gestation.

### Limitations and strengths

This study has several limitations. Although the randomized component of the intervention study was broken, the measures of physical fitness, pregnancy and birth outcomes were objective and unbiased. As a secondary analysis of the GESTAFIT study, it was not originally powered for these questions; however, the significant findings of objective measures support the presented physiological changes. Secondly, there are no validated tests for the evaluation of physical fitness in pregnant women, therefore we have used those that have been used in previous research with pregnant women [26,27,60]. Although the pregnant abdomen could be a mechanical barrier during the chair-stand test and the use of submaximal tests for the analysis of CRF may involve measurement error when estimating VO2max, there are few, if any, validated alternatives for assessing physical fitness parameters during pregnancy. Finally, our sample is relatively small and we have missing data due to various participant reasons. This study also has several strengths. Firstly, to the best of our knowledge, this is the first study providing a comprehensive examination of the association of objectively measured physical fitness indicators during pregnancy with maternal and neonatal outcomes. Secondly, paired samples were obtained (venous and arterial umbilical cord blood) which leads to a more reliable interpretation of the results. Thirdly, the gas measurement in arterial umbilical cord blood is a gold standard in the determination of the acid-base status in the neonate.

## Conclusions

Based on our results, women with greater upper-body muscle strength during pregnancy have neonates with higher birth weight. Likewise, greater CRF and flexibility during pregnancy were related to enhanced gas values in umbilical artery and less frequent caesarean sections. However, further research is necessary to confirm these novel findings.

## Supporting information

S1 File (DOC)

S2 File (DOCX)

S1 Table Inclusion and exclusion criteria in the GESTAFIT Project. (DOCX)

S2 Table Maternal physical fitness change from 16^th^ to 34^th^ gestational week. (DOCX)
