# Correction: Association of objectively measured physical fitness during pregnancy with maternal and neonatal outcomes. The GESTAFIT Project

**DOI:** 10.1371/journal.pone.0231230

**Published:** 2020-04-01

**Authors:** Laura Baena-García, Irene Coll-Risco, Olga Ocón-Hernández, Lidia Romero-Gallardo, Pedro Acosta-Manzano, Linda May, Virginia A. Aparicio

The word “birth-related” is missing from the title. The correct title is: Association of objectively measured physical fitness during pregnancy with maternal and neonatal birth-related outcomes. The GESTAFIT Project.

The following information is missing from the Funding statement: Unit of Excellence on Exercise and Health (UCEES), and by the Junta de Andalucía, Consejería de Conocimiento, Investigación y Universidades, European Regional Development Fund (ERDF), ref. SOMM17/6107/UGR. The correct Funding statement is as follows: This study was part of VAA fellowship from the Andalucía Talent-Hub Program, launched by the Andalusian Knowledge Agency, co-funded by the European Union’s Seventh Framework Program, Marie Skłodowska-Curie actions (COFUND–Grant Agreement nº291780) and the Junta de Andalucía. ICR (grant number: FPU13/01993) was supported by the Spanish Ministry of Education. This study was also partially funded by the Regional Ministry of Health of the Junta de Andalucía (PI-0395-2016). This study was supported by the University of Granada Plan Propio de Investigación 2016- Excellence actions: Unit of Excellence on Exercise and Health (UCEES), and by the Junta de Andalucía, Consejería de Conocimiento, Investigación y Universidades, European Regional Development Fund (ERDF), ref. SOMM17/6107/UGR. The funders had no role in study design, data collection and analysis, decision to publish, or preparation of the manuscript.
