# Supplementary material for: Association of objectively measured physical fitness during pregnancy with maternal and neonatal outcomes. The GESTAFIT Project
Source: PLoS One. 2020 Feb 18;15(2):e0229079. doi: 10.1371/journal.pone.0229079 (PMC7028270; doi:10.1371/journal.pone.0229079)
Supplement: S1 File — (DOC) [file pone.0229079.s001.doc]

| **Fecha:** | **Código:** |
| --- | --- |

**ENCUESTA INICIAL**

A continuación le pedimos que conteste a una serie de preguntas que nos permitirán conocer algunos datos importantes. Lea atentamente y conteste con sinceridad cada una de las respuestas. Debe saber que no existen preguntas correctas ni incorrectas. Rodee con un círculo el número correspondiente a la respuesta.

**A.- DATOS SOCIODEMOGRÁFICOS**

1.- Por favor, indique su fecha de nacimiento

| DIA | MES | AÑO |
| --- | --- | --- |
|  |  |  |

2.- ¿Cuál es su estado civil?

| Casado/a | 1 |
| --- | --- |
| Soltero/a | 2 |
| Separado/a | 3 |
| Divorciado/a | 4 |
| Viudo/a | 5 |

3.- ¿Actualmente usted vive solo/a o acompañado/a?

Pasa a pregunta 5

| Solo/a | 1 |
| --- | --- |
| Acompañado/a | 2 |

4.- ¿Quién convive con usted?

|  | SI | NO |
| --- | --- | --- |
| Pareja | 1 | 2 |
| Hijos | 1 | 2 |
| Otros familiares | 1 | 2 |

5.- Si tiene hijos ¿podría indicar cuántos? ____hijos

6.- ¿Qué estudios tiene usted que haya completado?

| Sin Estudios | 1 |
| --- | --- |
| Primarios: Educación Primaria, EGB | 2 |
| Formación Profesional | 3 |
| Secundarios: Bachillerato, BUP, COU | 4 |
| Universitarios de Grado Medio | 5 |
| Universitarios de Grado Superior | 6 |

**B.-DATOS CLÍNICOS**

7. ¿Tiene alguna enfermedad diagnosticada (física o psicológica)?

| Sí | 1 | Continúe por bloque C – MESTRUACIÓN/ MENOPAUSIA |
| --- | --- | --- |
| No | 2 |

8.- De las siguientes enfermedades, indique aquellas que le han sido diagnosticadas por un/a médico/a.

|  | **SI** | **NO** |
| --- | --- | --- |
| Tensión alta | 1 | 2 |
| Infarto de miocardio | 1 | 2 |
| Otras enfermedades del corazón | 1 | 2 |
| Varices en las piernas | 1 | 2 |
| Artrosis, artritis o reumatismo | 1 | 2 |
| Dolor de espalda crónico (cervical) | 1 | 2 |
| Dolor de espalda crónico (lumbar) | 1 | 2 |
| Alergia crónica | 1 | 2 |
| Asma | 1 | 2 |
| Bronquitis crónica | 1 | 2 |
| Diabetes | 1 | 2 |
| Úlcera de estómago o duodeno | 1 | 2 |
| Incontinencia urinaria | 1 | 2 |
| Colesterol alto | 1 | 2 |
| Cataratas | 1 | 2 |
| Problemas crónicos de la piel | 1 | 2 |
| Estreñimiento crónico | 1 | 2 |
| Depresión, ansiedad u otros trastornos mentales | 1 | 2 |
| Fibromialgia | 1 | 2 |
| Embolia | 1 | 2 |
| Migraña o dolor de cabeza frecuente | 1 | 2 |
| Hemorroides | 1 | 2 |
| Tumores malignos | 1 | 2 |
| Osteoporosis | 1 | 2 |
| Anemia | 1 | 2 |
| Problemas de tiroides | 1 | 2 |
| ¿Alguna otra enfermedad crónica? ………………. | 1 | 2 |

9.- Durante los **últimos doce meses**, ¿esa/s enfermedad/es o problema/s de salud, le han limitado de alguna forma sus actividades habituales?

| Sí | 1 |
| --- | --- |
| No | 2 |

**C.- MENSTRUACIÓN / MENOPAUSIA**

10.- ¿A qué edad tuvo su primera menstruación? A los _______ años

11- ¿Cuántos **hijos** ha tenido? _____ hijos

12- ¿Cuántos **abortos** ha tenido? _____ abortos

13.- ¿Ha tomado **anteriormente** anticonceptivos hormonales?

13.2 ¿Cuánto tiempo estuvo tomándolos?

| Años | Meses |
| --- | --- |
|  |  |

| Sí | 1 |
| --- | --- |
| No | 0 |

14.- ¿De qué tipo? ___________

**E.- TRABAJO**

15.- ¿Cuál es su dedicación y/o actividad laboral **actual**?

| Trabajo a tiempo completo remunerado | 1 |  |
| --- | --- | --- |
| Trabajo a tiempo parcial remunerado | 2 |  |
| Trabajo doméstico no remunerado (exclusivamente) | 3 | Si ha contestado alguna de estas respuestas, pase a la pregunta 24 |
| Estudiante | 4 |
| Retirado/jubilado | 5 |
| Retirado/jubilado por incapacidad laboral | 6 |
| Baja laboral por enfermedad | 7 |
| En paro | 8 |

16.- ¿Podría indicar exactamente qué tipo de trabajo desempeña en la **actualidad?**

| Dirección de empresas y administraciones públicas | 1 |
| --- | --- |
| Técnicos y profesionales científicos e intelectuales | 2 |
| Técnicos y profesionales de apoyo | 3 |
| Empleados de tipo administrativo | 4 |
| Trabajadores de servicio de restauración, personales, protección y vendedores de comercio | 5 |
| Trabajadores cualificados en agricultura y en la pesca | 6 |
| Artesanos y trabajadores cualificados de industrias manufactureras, construcción, y minería, excepto operadores de instalación y maquinaria | 7 |
| Operadores instalaciones y maquinaria, y montadores | 8 |
| Trabajadores no cualificados | 9 |
| Fuerzas armadas | 10 |
| Otra (indicar): ___________ | 13 |

17.- ¿Podría indicar en una escala de 0 a 10 su nivel de SATISFACCIÓN con el trabajo actual?

| 1 | 2 | 3 | 4 | 5 | 6 | 7 | 8 | 9 | 10 |
| --- | --- | --- | --- | --- | --- | --- | --- | --- | --- |

Nada satisfecho Muy satisfecho

**FIN DEL TEST**
