# Supplementary material for: Association of objectively measured physical fitness during pregnancy with maternal and neonatal outcomes. The GESTAFIT Project
Source: PLoS One. 2020 Feb 18;15(2):e0229079. doi: 10.1371/journal.pone.0229079 (PMC7028270; doi:10.1371/journal.pone.0229079)
Supplement: S2 File — (DOCX) [file pone.0229079.s002.docx]

| **Date:** | **Code:** |
| --- | --- |

**INITIAL SURVEY**

Here we ask you to answer a series of questions that will allow us to know some important information. Read carefully and answer honestly each of the answers. You should be aware that there are no right or wrong questions. Circle the number corresponding to the answer.

**A.- SOCIODEMOGRAPHIC DATA**

1.- Please indicate your date of birth

| DAY | MONTH | YEAR |
| --- | --- | --- |
|  |  |  |

2.- What is your marital status?

| Married | 1 |
| --- | --- |
| Single | 2 |
| Separated | 3 |
| Divorced | 4 |
| Widow | 5 |

3.- Do you currently live alone or with someone?

| Alone | 1 |
| --- | --- |
| With someone | 2 |

Go to question 5

4.- Who lives with you?

|  | YES | NO |
| --- | --- | --- |
| Partner | 1 | 2 |
| Children | 1 | 2 |
| Other familiy | 1 | 2 |

5.- If you have children, could you indicate how many? ___ children

6.- What studies have you finished?

| Without studies | 1 |
| --- | --- |
| Elementary school | 2 |
| Professional training | 3 |
| High School | 4 |
| University degree (medium level) | 5 |
| University degree (high level) | 6 |

**B.-CLINICAL DATA**

7. Do you have any diagnosed illness (physical or psychological)?

| Yes | 1 | Continue on block C - MESTRUATION / MENOPAUSE |
| --- | --- | --- |
| No | 2 |  |

8.- Of the following diseases, indicate those that have been diagnosed by a doctor.

|  | **YES** | **NO** |
| --- | --- | --- |
| Hypertension | 1 | 2 |
| Heart attack | 1 | 2 |
| Other heart diseases | 1 | 2 |
| Varicose veins in the legs | 1 | 2 |
| Osteoarthritis, arthritis or rheumatism | 1 | 2 |
| Chronic back pain (cervical) | 1 | 2 |
| Chronic back pain (lumbar) | 1 | 2 |
| Chronic allergy | 1 | 2 |
| Asthma | 1 | 2 |
| Chronic bronchitis | 1 | 2 |
| Diabetes | 1 | 2 |
| Stomach or duodenal ulcer | 1 | 2 |
| Urinary incontinence | 1 | 2 |
| High cholesterol | 1 | 2 |
| Waterfalls | 1 | 2 |
| Chronic skin problems | 1 | 2 |
| Chronic constipation | 1 | 2 |
| Depression, anxiety or other mental disorders | 1 | 2 |
| Fibromyalgia | 1 | 2 |
| Embolism | 1 | 2 |
| Migraine or frequent headache | 1 | 2 |
| Hemorrhoids | 1 | 2 |
| Malignant tumors | 1 | 2 |
| Osteoporosis | 1 | 2 |
| Anemia | 1 | 2 |
| Thyroid problems | 1 | 2 |
| Another cronic disease? | 1 | 2 |

9.- During the LAST TWELVE MONTHS, has that disease / s or health problem / s limited your usual activities in any way?

| Yes | 1 |
| --- | --- |
| No | 2 |

**C.- MENSTRUATION / MENOPAUSE**

10.- At what age was your first menstruation? _______ years old

11- How many children have you had? _____ children

12- How many abortions have you had? _____ abortions

13.- Have you previously taken hormonal contraceptives?

13.2 How long were you taking them?

| Years | Months |
| --- | --- |
|  |  |

| Yes | 1 |
| --- | --- |
| No | 0 |

14.- What type? ___________

**D.- TRABAJO**

15.- What is your dedication and / or CURRENT work activity?

| Full-time paid work | 1 |  |
| --- | --- | --- |
| Part-time paid work | 2 |  |
| Unpaid domestic work (exclusively) | 3 | If you have answered any of these answers, go to question 24 |
| student | 4 |  |
| Retired / retired | 5 |  |
| Retired / retired due to work disability | 6 |  |
| Occupational sick leave | 7 |  |
| Unemployed | 8 |  |

16.- Could you indicate exactly what type of work you are currently doing?

| Management of companies and public administrations | 1 |
| --- | --- |
| Scientific and intellectual technicians and professionals | 2 |
| Technicians and support professionals | 3 |
| Administrative employees | 4 |
| Catering service workers, personnel, protection and trade sellers | 5 |
| Skilled workers in agriculture and fishing | 6 |
| Craftsmen and skilled workers in manufacturing, construction, and mining industries, except installation and machinery operators | 7 |
| Plant and machinery operators and assemblers | 8 |
| Unskilled workers | 9 |
| Armed forces | 10 |
| Other (indicate): ___________ | 13 |

17.- Could you indicate on a scale of 0 to 10 your level of SATISFACTION with the current job?

| 1 | 2 | 3 | 4 | 5 | 6 | 7 | 8 | 9 | 10 |
| --- | --- | --- | --- | --- | --- | --- | --- | --- | --- |

Not at all satisfied Very satisfied

**END OF TEST**
