# Supplementary material for: Association of objectively measured physical fitness during pregnancy with maternal and neonatal outcomes. The GESTAFIT Project
Source: PLoS One. 2020 Feb 18;15(2):e0229079. doi: 10.1371/journal.pone.0229079 (PMC7028270; doi:10.1371/journal.pone.0229079)
Supplement: S1 Table — (DOCX) [file pone.0229079.s003.docx]

**Table S1.** Inclusion and exclusion criteria in the GESTAFIT project.

| ***Inclusion criteria*** |
| --- |
| - Pregnant women aged 25-40 years old with a normal pregnancy course. |
| - Answering “no” to all questions on the PARmed-X for pregnancy.  - Being able to walk without assistance.  - Being able to read and write properly.  - Informed consent: Being capable and willing to provide written consent.  *In addition, specific inclusion criteria for data analysis are: pregnancy with single foetus, spontaneous or instrumental vaginal birth, and caesarean without maternofoetal pathology (or any other indication that does not involve maternofoetal risk, such as disproportion, or non-cephalic presentation). |
| ***Exclusion criteria*** |
| - Acute or terminal illness.  - Malnutrition.  - Inability to conduct tests for assessing physical fitness or exercise during pregnancy.  - Underweight.  - Pregnancy risk factors (such as hypertension, type 2 diabetes, etc.).  - Multiple pregnancy.  - Chromosopathy or foetal malformations.  - Uterine growth restriction.  - Foetal death.  - Upper or lower extremity fracture in the past 3 months.  - Presence of neuromuscular disease or drugs affecting neuromuscular function.  - Being registered in another exercise program.  - Doing more than 300 minutes of at least moderate physical activity per week.  - Unwillingness either to complete the study requirements or to be randomised into the control or intervention group. |
