# Supplementary material for: Association of objectively measured physical fitness during pregnancy with maternal and neonatal outcomes. The GESTAFIT Project
Source: PLoS One. 2020 Feb 18;15(2):e0229079. doi: 10.1371/journal.pone.0229079 (PMC7028270; doi:10.1371/journal.pone.0229079)
Supplement: S2 Table — (DOCX) [file pone.0229079.s004.docx]

|  | **Mean change in physical fitness (SD)** | **95% CI** | **p** |
| --- | --- | --- | --- |
| Flexibility (Back-scratch test), cm | -0.398 (2.68) | (-0.883, -0.086) | 0.106 |
| Upper-body muscle strength (Hand-grip test), Kg | -0.261 (2.96) | (-0.793, 0.270) | 0.333 |
| Lower-body muscle strength (Chair-stand test), rep | -0.169 (2.51) | (-0.791, 0.453) | 0.589 |
| Cardiorrespiratory fitness (Bruce test), mL/(kg·min) | -4.14 (5.07) | (-5.272, -3.014) | <0.001 |

**Table S2.** Maternal physical fitness change from 16^th^ to 34^th^ gestational week

SD, standard desviation; CI, Confidence Interval
